# Length-based separation of *Arthrospira* (*Spirulina*) *platensis* trichomes via the self-alignment effect of helical filaments in a straight microchannel

**DOI:** 10.1038/s41378-026-01302-4

**Published:** 2026-05-12

**Authors:** Kodai Hara, Akihiro Isozaki

**Affiliations:** Department of Mechanical Engineering, Ritsumeikan University, Kusatsu, Shiga Japan

**Keywords:** Electrical and electronic engineering, Chemistry

## Abstract

Shape-based separation of biological particles has elucidated cellular morphology and function. However, length-based sorting of helical-shaped microorganisms, such as *Arthrospira* (*Spirulina*) *platensis*, in microfluidic platforms has not yet been achieved. Here, we report a passive, length-based sorting method for *A. platensis* trichomes using a straight microchannel that exploits a self-alignment effect of helical filaments. In our design, trichomes flowed through a straight microchannel narrower than their average length and entered a downstream expanding channel with five outlets. High-speed camera-based observation revealed that shorter trichomes predominantly aligned near the channel walls, whereas longer trichomes spanned the channel width. These alignment tendencies, termed the self-alignment effect, were flow-rate dependent and most prominent at a Reynolds number of 40. Thus, short and long trichomes were guided to different outlet positions. Quantitative analysis showed that at a threshold length of 300 µm, sorting purities of 77% and 84% were achieved for short and long trichomes, respectively. This demonstrates effective, label-free separation of multicellular cyanobacterial filaments based on length in a simple microfluidic geometry. As trichome length reflects the growth stage and environmental response, this method offers a promising platform for studying physiological heterogeneity in *A. platensis* populations. Furthermore, it can facilitate applications in biotechnology and materials science, where precise selection of filamentous morphologies is desired. This principle can also be applied to other microorganisms or flexible helical structures. The observed self-alignment effect may become a new paradigm in passive microscale sorting.

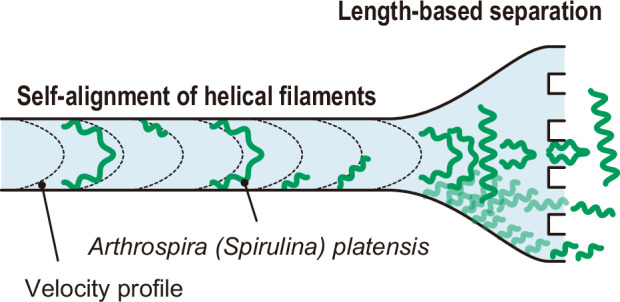

## Introduction

The microfluidic-based sorting of photosynthetic microorganisms has attracted increasing attention because of the high-throughput capabilities of microfluidic platforms^1–3^ and the biological and industrial potential of these organisms in fields such as food production, renewable energy, carbon fixation, and environmental remediation^4–7^. Among the various sorting techniques, fluorescence-activated cell sorting is a widely used, commercially available method for sorting microalgae and other photosynthetic cells^8–10^. To expand the versatility of sorting, multiple alternative methods have been developed. In particular, the diverse morphologies of photosynthetic microorganisms have been used as indicators of physiological or functional states. For example, acoustic-based cell sorting leverages size-dependent acoustic forces^11^, enabling morphology-based separation^12^. Image-activated cell sorting can visualise and utilise morphological features directly^13,14^. For example, *Chlamydomonas reinhardtii* cells with defective carbon-concentrating mechanisms have been successfully sorted using this method^15,16^. Inertial focusing-based cell sorting^17^ using a straight channel enables morphology-sensitive separation, where *Euglena gracilis* cells are sorted on the basis of the aspect ratio^18^. Inertial focus-based cell sorting via a spiral channel enables the separation of *Tetraselmis suecica* cells from *Phaeodactylum tricornutum* cells^19^. Viscoelastic focusing-based sorting enables length-based bacterial sorting^20,21^.

Despite these advancements, to our knowledge, no prior work has demonstrated the passive, length-based sorting of *Arthrospira* (*Spirulina*) *platensis* trichomes. *A. platensis* is a photosynthetic cyanobacterium often referred to as a microalga in industrial contexts. It forms characteristic helical-shaped multicellular filaments with diameters of ~10 µm and lengths ranging from ~100 to ~600 µm. Moreover, it grows via cell division along its trichome, followed by occasional fragmentation into shorter filaments. It is widely used in applications such as food^22,23^, biofuel^24,25^, biodegradable materials/bioplastics^26,27^, and bioremediation^28^. Owing to its unique helical morphology, its physical characteristics, such as length, bending stiffness, elastic modulus, and motility, have been studied^29,30^. Among them, the length of the trichomes is considered the most fundamental morphological parameter of *A. platensis*, as it reflects the growth stage and fragmentation dynamics of *A. platensis*^31^. For example, prior work has reported that unusually short *A. platensis* trichomes (<250 µm) exhibit distinct length-dependent gliding motility^30^. However, sorting such morphologically significant organisms remains challenging, especially because manipulating long, flexible structures in microfluidic channels is technically challenging.

**Fig. 1:**
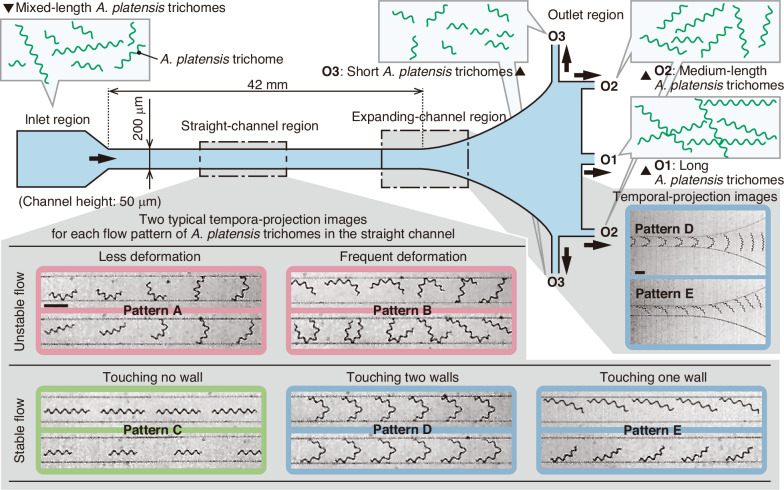
Length-based separation of *A. platensis* trichomes using a straight microchannel with an expanding channel downstream. The microfluidic device consists of the inlet, straight-channel, expanding-channel, and outlet regions. *A. platensis* trichomes of mixed lengths are inserted into the channel (see the inset schematic of the inlet region), flow in the straight channel, and are separated into five outlets depending on their length. Typical temporal-projection high-speed camera images of individual *A. platensis* trichomes flowing in the straight microchannel, showing that the flowing behaviour can be categorized into five patterns: Pattern A, in which trichomes unstably flow with less deformation; Pattern B, in which trichomes unstably flow with frequent deformation; Pattern C, in which trichomes stably flow without touching channel walls; Pattern D, in which trichomes stably flow with touching two walls; and Pattern E, in which trichomes stably flow with touching one wall. Typical temporal-projection high-speed camera images of individual *A. platensis* trichomes flowing in the expanding-channel region, indicating that trichomes deflect depending on the behaviour in the straight channel. Scale bars; 200 µm. O1, Outlet 1; O2, Outlet 2; and O3, Outlet 3. Here, we defined these outlet names considering the symmetrical configuration of the channel

In this study, we propose and experimentally demonstrate the length-based separation of *A. platensis* trichomes via the self-alignment effect of helical filaments observed in confined microchannel flow (Fig. 1). This effect causes trichomes to deflect in different directions in a downstream expanding-channel region, depending on their length. Specifically, we designed and fabricated a microfluidic device consisting of a long straight microchannel narrower than the average trichome length. Interestingly, within this confined geometry, we found via high-speed imaging that many trichomes tended to align closely with the flow velocity profile, and shorter and longer trichomes remained near the channel walls and spanned the width of the channel, respectively. We utilised this flow-induced self-alignment effect of helical filaments to achieve passive, length-based sorting by incorporating five outlets downstream of the channel. High-speed camera observations revealed that the purities of the sorting experiments were expected to be >85% for both short and long trichomes when a threshold of 300 µm, which is 1.5 times larger than the width of the straight channel, was applied. Furthermore, we assessed the aliquots collected from the outlets, revealing >77% sorting purities. Our experiments demonstrate that this approach enables effective separation of *A. platensis* trichomes on the basis of length in a straight microchannel.

## Results and discussion

### Flow pattern analysis of trichomes in straight and expanding microchannels

We fabricated the microchannel (see Fig. 1 for the physical dimensions of the channel) and used it to observe and analyse the behaviours of *A. platensis* trichomes in the straight-channel region and the expanding-channel region (see “Materials and methods” for the details of the expanding-channel functionality). As shown in the inset temporal-projection images for the straight-channel region, the flowing pattern can be categorized into five patterns: (1) trichomes unstably flow with less deformation (Pattern A); (2) trichomes unstably flow with frequent deformation (Pattern B); (3) trichomes stably flow without touching walls (Pattern C); (4) trichomes stably flow with touching two walls (Pattern D); and (5) trichomes stably flow touching one wall (Pattern E) (see Movie S1 for an example of a video we analysed). Notably, trichomes flowing with Pattern C aligned with the centre line, whereas trichomes flowing with Pattern D exhibited C-shaped bending; moreover, trichomes flowing with Pattern E flowed close to a channel wall with a finite angle (neither perpendicular nor parallel to the flow direction). In this study, we refer to the latter patterns (e.g. Patterns D and E) as the “self-alignment effect of helical filaments”. These pattern differences determine the outlet that the trichomes flowed into, as shown in the inset temporal-projection images for the expanding-channel region (see Movie S2 for typical filaments flowing with Patterns D and E).

**Fig. 2:**
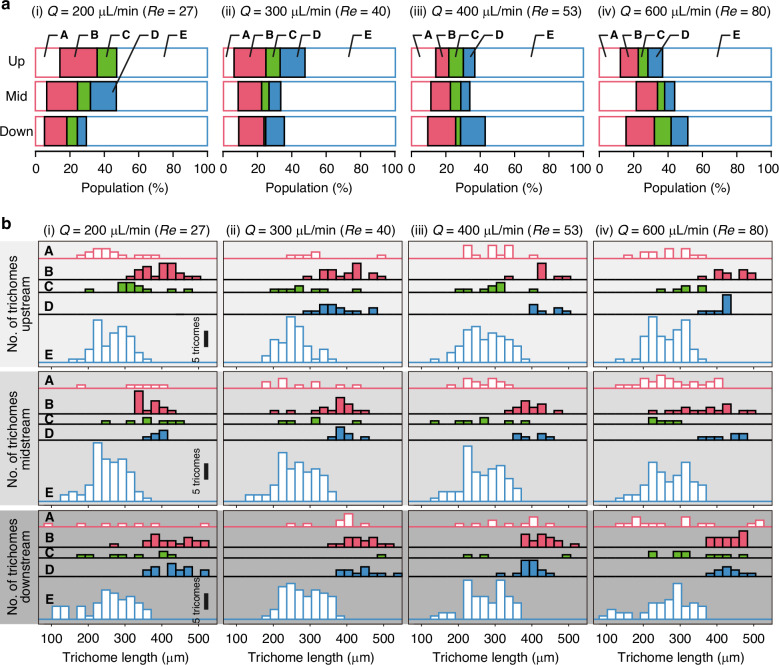
Quantitative analysis of the behaviour of flowing *A. platensis* trichomes. The behaviours were analysed at the upstream, midstream, and downstream positions of the straight microchannel under various flow rates. The analysed window positions of the upstream, midstream, and downstream regions are 0-6, 17-23, and 37-42 mm from the entrance of the straight channel, respectively. Patterns A–E are defined in Fig. 1. **a** Population of the flow patterns. Up, upstream; Mid, midstream; Down, downstream. **b** *A. platensis* trichome length distributions. The distribution pattern differs by flow patterns. The numbers of data for the flow rate of 200 µL/min at the upstream, midstream, and downstream positions are 106, 115, and 106, respectively; for the flow rate of 300 µL/min at the upstream, midstream, and downstream positions, the numbers are 97, 117, and 102, respectively; for the flow rate of 400 µL/min at the upstream, midstream, and downstream positions, the numbers are 106, 115, and 105, respectively; and for the flow rate of 600 µL/min at the upstream, midstream, and downstream positions, the numbers are 106, 121, and 103, respectively. *Q*, flow rate

We quantitatively analysed the behaviour of *A. platensis* trichomes in the microchannel with various flow rates, i.e. various Reynolds numbers (see “Materials and methods” for the definition of the Reynolds number), upstream, midstream, and downstream of the straight microchannel. The analysis results of the populations of each pattern in the straight-microchannel region are shown in Fig. 2a. Although the trichome length distributions vary slightly among the stacked bar graphs of the population distributions due to sampling variability (Fig. S1), the results still offer multiple insights. Specifically, these results suggest that Pattern E was the most frequently observed pattern across all the conditions and regions. The populations of self-aligned trichomes (Patterns D and E) increased flowing upstream to downstream at low Reynolds numbers (*Re* = 27, 40, and 53; Fig. 2a-ii, and 2a-iii, respectively), suggesting that the self-aligned orientation is energetically stable. The populations of unstably flowing (Patterns A and B) trichomes increased from upstream to downstream at Reynolds numbers of 53 (Fig. 2a-iii) and 80 (Fig. 2a-iv), indicating that high-Reynolds-number flow breaks self-aligned stable flow; therefore, the onset location of this increase depends on the Reynolds number. Specifically, at the Reynolds number of 53 (Fig. 2a-iii), the increase occurs between midstream and downstream, whereas at the Reynolds number of 80 (Fig. 2a-iv), it occurs between upstream and midstream. This shift suggests that higher-Reynolds-number flow disrupts self-aligned stable flow earlier along the channel.

The trichome length dependency of the population is depicted in Fig. 2b. By focusing on the low Reynolds number conditions (*Re* = 27 and 40; Fig. 2b-ii, respectively), we confirmed that the major flow pattern of short trichomes (<~300 µm) was Pattern E. In comparison, the population of long trichomes (>~300 µm) was a mixture of the other patterns (Patterns A-D). In particular, at a Reynolds number of 40 (Fig. 2b-ii), short trichomes changed their flowing patterns from Pattern A or C upstream or midstream to Pattern E downstream.

**Fig. 3:**
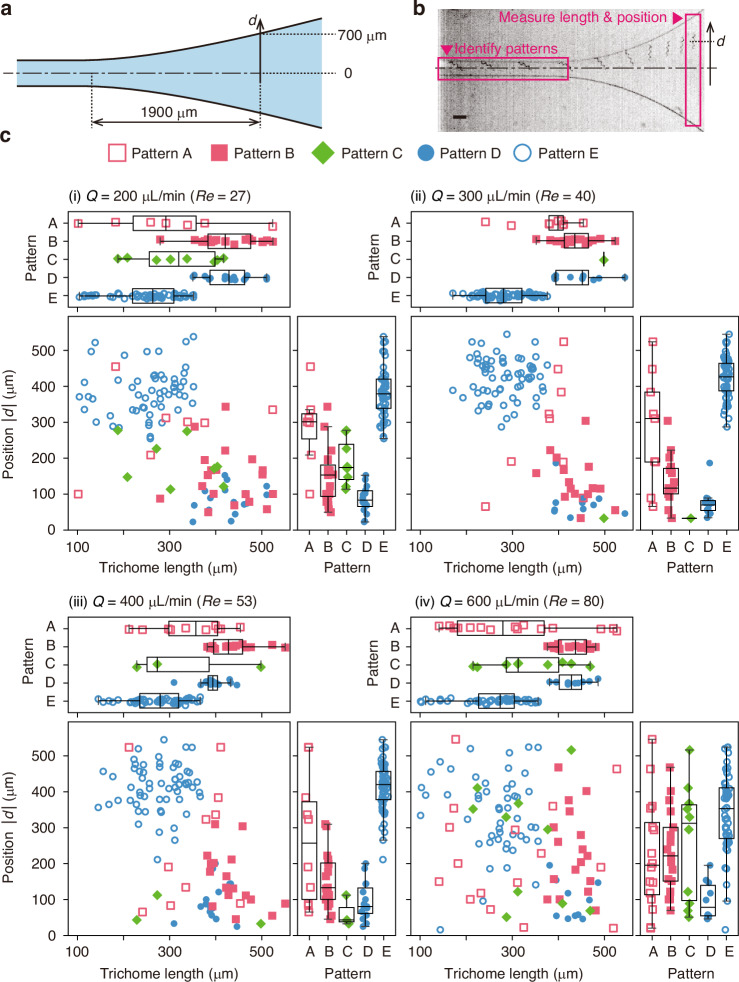
Relation between the trichome length and the trichome position *d* in the expanding-channel region labelled with flow patterns in the straight channel. Patterns A–E are defined in Fig. 1. **a** Definition of position *d* used in this figure. The trichome positions were measured 1900 µm downstream from the entrance of the expanding-channel region. **b** Typical temporal-projection high-speed camera image of an *A. platensis* trichome. The flow pattern is identified in the left-box region, and the trichome length and position *d* are measured in the right-box region. Scale bar, 200 µm. **c** Scatter plots and box plots of the trichome length and the trichome position *d* in the expanding-channel region. Boxplots were generated using Tukey’s method, in which the box represents the interquartile range (IQR) between the 25th percentile (Q1) and the 75th percentile (Q3). Whiskers extend to the most extreme data points within 1.5 × IQR from the quartiles, and data beyond these limits are considered outliers and plotted individually. The numbers of data are described in the caption of Fig. 2. *Q*, flow rate

The correlations between the trichome length, the position of the trichome in the expanding-channel region, and the flow pattern are shown in Fig. 3. The definition of position *d* used in this figure is shown in Fig. 3a. As shown in Fig. 3b, the flow pattern of each trichome is identified in the left-box region, and the length and position of each trichome are measured in the right-box region. Figure 3c shows the scatter plots and box plots to quantitively discuss the behaviour of trichomes in the expanding-channel region. These scatter plots show two main clusters around the left upper region and right bottom region, which are noticeable when the Reynolds number is 40 (Fig. 3b-ii). This characteristic is good for achieving length-based trichome sorting. The box plots for *Re* = 40 provide insight into the separation mechanism, i.e. a trichome’s flow pattern in the straight-channel region determines its deflection direction in the expanding-channel region, thereby determining the outlet it exits through (summarised in Table 1). Specifically, *A. platensis* trichomes that flow with Pattern A migrate randomly in a lateral direction to the flow direction and then flow randomly into the expanding-channel region. This would lead to random flow into five outlets. *A. platensis* trichomes flowing with Patterns B and D tend to move towards the central outlet (O1; see Fig. 1 for the identification of outlet O1). This might be because these trichomes cover the entire width of the straight microchannel. Trichomes that flow with Pattern C tend to maintain their position, moving towards the central outlet. On the other hand, trichomes flowing with Pattern E flow close to the wall in the straight microchannel and maintain their position in the expanding-channel region, leading to flow into the side outlets (O3; see Fig. 1 for the identification of outlet O3).

**Table 1:** Outlet assignment trends for each flow pattern (*Re* = 40)

Pattern	Outlet
A	Random
B	Centre outlet (O1)
C	Centre outlet (O1)
D	Centre outlet (O1)
E	Side outlet (O3)

Outlet labels (O1-O3) are defined in Fig. 1

### High-speed camera-based evaluation of length-based trichome separation

**Fig. 4:**
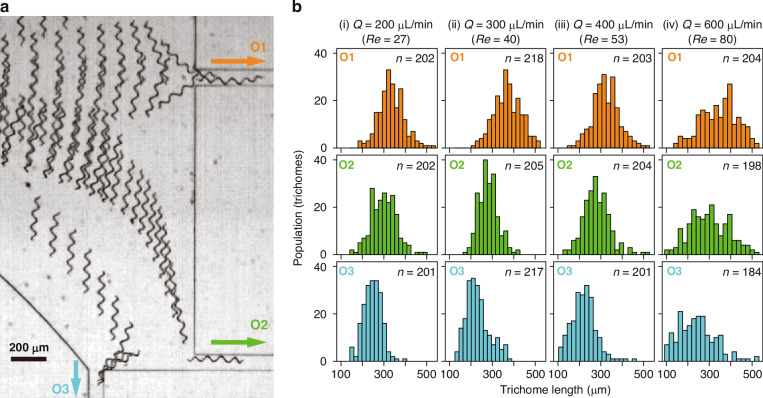
Length-based separation of *A. platensis* trichomes. **a** Typical temporal-projection high-speed camera image at the outlets, showing that *A. platensis* trichomes with different length are more likely to exit from different outlets: longer shaped and shorter shaped trichomes exit from outlets centreline and closer to the sidewall, respectively. The flow rate when the video recorder was set to 300 µL/min. **b** A comparison of the population for *A. platensis* trichomes flowing into each outlet with different length. O1, Outlet 1; O2, Outlet 2; O3, Outlet 3. *Q*, flow rate

We experimentally demonstrated *A. platensis* trichome separation and evaluated it via high-speed camera-based analysis of trichomes flowing into five outlets (one O1, two O2s, and two O3s, as defined in Fig. 1). Figure 4a and Movie S3 depict typical behaviours of trichomes in the outlet region, showing that long trichomes, middle trichomes, and short trichomes flow into O1, O2, and O3, respectively. To further evaluate the potential of *A. platensis* trichome sorting on the basis of length, we quantified the length and number of *A. platensis* trichomes that flow into each outlet. As shown in Fig. 4b, longer *A. platensis* trichomes tended to preferentially flow into O1, whereas shorter trichomes tended to flow into O3. This separation trend is particularly remarkable when the Reynolds number is 40 (Fig. 4b-ii); however, the trend is eliminated when the Reynolds number is 80 (Fig. 4b-iv). These results are consistent with those discussed in Fig. 3.

**Fig. 5:**
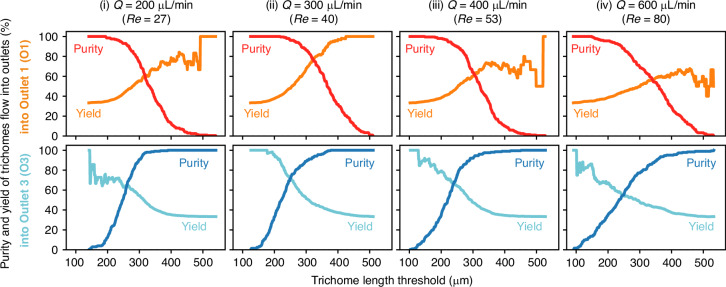
Performance of the length-based separation of *A. platensis* trichomes. The purities and yields are calculated as a function of the trichome-length threshold. The calculation was performed assuming that an equal number of trichomes flowed into each outlet. *Q*, flow rate

To further evaluate the potential of our separation method, we calculated the purities and yields of the separation based on the data shown in Fig. 4b. Specifically, we calculated the values of O1 and O3 as a function of the trichome-length threshold on the basis of two assumptions: (1) O1 and O3 are used to sort long and short trichomes, respectively; (2) trichomes flow into each outlet equally. The details of the calculation are provided in the “Materials and methods” section. Here, we consider that O2 works as a buffer to separate long and short trichomes. This concept reduces the sorting yield but increases the purity. As shown in Fig. 5, the purity and yield both vary depending on the flow rate (Reynolds number), and their values have a trade-off relationship. Among the flow rates tested, the highest purity for the short-trichome separation using O1 was achieved at a flow rate of 300 µL/min (*Re* = 40, Fig. 5(ii)), whereas the highest purity for the long-trichome separation using O3 was achieved at a Reynolds number of 53 (Fig. 5(iii)). Specifically, these separation purities at a threshold of 300 µm for short and long trichome separation were 86% and 92%, respectively. This proof-of-concept experimental validation demonstrates the potential of our microchannel for the effective sorting of *A. platensis* on the basis of trichome length.

### Demonstration of length-based trichome sorting

**Fig. 6:**
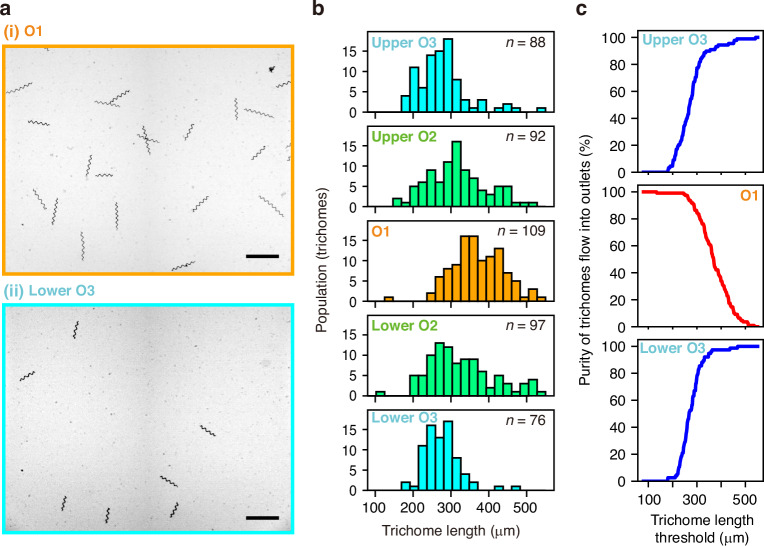
Length-based sorting demonstration. **a** Representative microscopic photos of aliquots collected from O1 and lower O3. Scale bars, 500 µm. **b** A comparison of the population of *A. platensis* trichomes collected from each outlet with different lengths. **c** Purities calculated as a function of the trichome-length threshold. A 200-µm-wide microchannel device was used. The Reynolds number in the straight channel was set at 40. O1, Outlet 1; O2, Outlet 2; O3, Outlet 3

We demonstrated the length-based sorting of *A. platensis* trichomes and evaluated the sorting performance using trichomes collected from each outlet. For this sorting demonstration, we used a microfluidic device with a 200-µm-wide straight channel and flowed a trichome suspension at *Re* = 40. After the sorting, we analysed aliquots of the collected fractions from the five outlets. Aliquots of the collected samples were deposited on glass slides and observed under a microscope. Representative photos from O1 and lower O3, showing that the lengths of collected trichomes in O1 are longer than those in lower O3, are shown in Fig. 6a. As shown in Fig. 6b, our quantitative assessments support that the outer outlets (two O3s) collected shorter trichomes, whereas the centre outlet (O1) collected longer trichomes. We calculated the purities of two O3s and O1 (Fig. 6c), and these results are consistent with the data shown in Fig. 5. Specifically, when the length threshold was set at 300 µm, the purities of the upper O3 and the lower O3 were 77.3% and 77.6%, respectively, and that of O1 was 84.4%. Here, we did not report yield because the analysis was performed on aliquots rather than the entire collected volume, which prevents a reliable quantification of the total number of trichomes recovered from each outlet.

### Potential mechanisms of the self-alignment effect

To gain mechanistic insight into the self-alignment effect of the helical filaments, we examined trichome flow behaviours over a wide range of Reynolds numbers (*Re* = 0.4–-400) and channel widths (*w* = 150–300 µm). We hypothesized that the shear-rate distribution in the channel cross-section plays a central role in determining the observed flow patterns. We therefore first performed finite-element method-based simulations to estimate velocity and shear-rate profiles in straight channels and then sought to interpret the experimentally observed trichome behaviours.

**Fig. 7:**
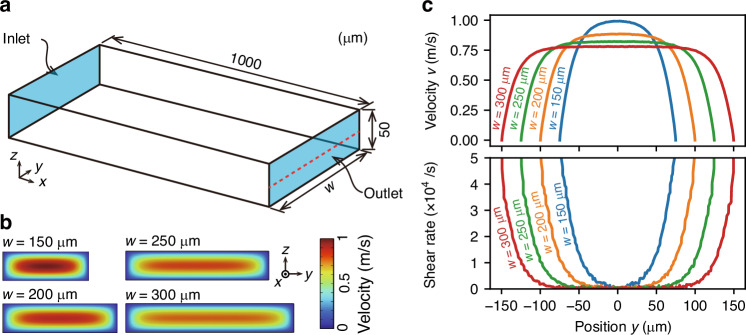
Flow simulation in the straight microchannel. **a** Three-dimensional simulation model. **b** Flow velocity distributions on the outlet cross-section with various channel widths at *Re* = 40. **c** Flow velocity and shear-rate profiles on the centreline (the red dashed line shown in **a**) of the outlet cross-section with various channel widths *w*, at *Re* = 40

The three-dimensional simulation model we constructed, which is a cuboid with a length of 200 µm, height of 50 µm, and variable width *w*, is shown in Fig. 7a. The detailed settings are described in the “Materials and methods” section. Figure 7b shows the velocity distributions on the outlet cross-section for different channel widths, and Fig. 7c shows the velocity and shear-rate profiles along the centreline on the outlet cross-section (red dashed line in Fig. 7a). Here, the wall-normal shear rate is defined as $\left|\partial {v}_{x}/\partial y\right|$, where ${v}_{x}$ denotes the streamwise velocity as a function of *y*. These results confirm that the shape of the flow velocity profile varies drastically with the channel aspect ratio. For instance, a parabolic-like flow profile is observed for *w* = 150 µm, whereas the profile becomes plug-like as the channel width increases. Interestingly, the C-like configuration observed for Pattern D in the 200-µm-wide channel (see Fig. 1) resembles the corresponding flow velocity profile shown in Fig. 7c. This finding suggests that Pattern D can be rationalized by the shear-rate distribution. Specifically, a trichome experiences non-uniform hydrodynamic loading along the trichome to generate bending. As a result, sufficiently long trichomes are deformed towards curved configurations. In contrast, although Pattern E trichomes look like a part of the flow velocity profile, Pattern E is not fully accounted for by the shear-rate distribution alone, suggesting that additional effects (e.g. axial extensibility, helical geometry, and/or finite inertia effects) may contribute.

**Fig. 8:**
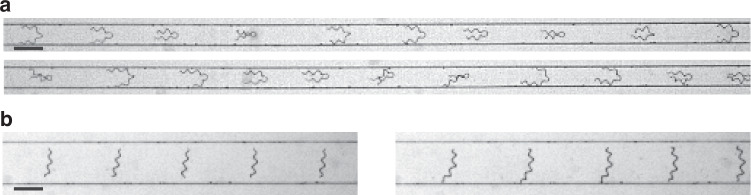
Typical temporal-projection high-speed camera images of individual *A. platensis* trichomes flowing in straight channels, exhibiting different flowing behaviours from those shown in Fig. 1. **a** In the 150-µm-wide channel: Pattern F, in which trichomes exhibit oscillatory flow. **b** In the 300-µm-wide channel: Pattern G, in which trichomes flow without bending. Scale bars; 200 µm

Next, we performed experiments over a wide range of Reynolds numbers from 0.4 to 400 and channel widths from 150 to 300 µm. Through these experiments, we found two patterns that are different from Patterns A-E. First, we found trichomes exhibiting oscillatory flow (Pattern F, see Fig. 8a) when we observed them flowing in the 150-µm-wide channel (Movie S4). This flow pattern can be interpreted as a pattern with a sharper bending angle than that in Pattern D. Next, we found trichomes flowing oriented perpendicular to the flow direction with minimal bending (Pattern G, see Fig. 8b) when we observed them flowing in the 300-µm-wide channel (Movie S5). This flow pattern can be interpreted as a pattern with a larger bending angle (~π) than that in Pattern D. Interestingly, both patterns (Patterns F and G) are consistent with the flow velocity profiles shown in Fig. 7c.

**Fig. 9:**
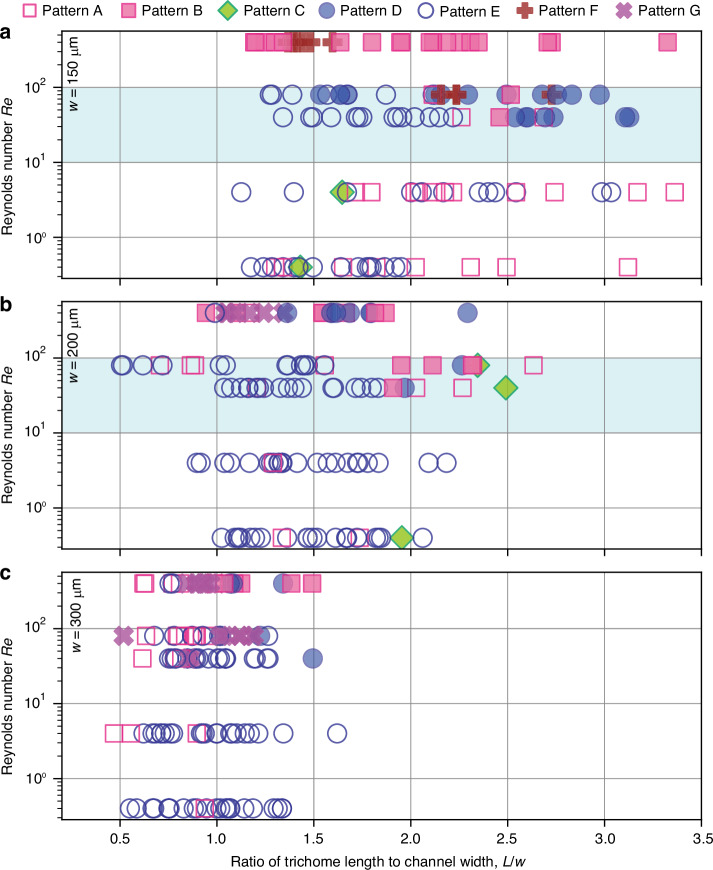
Map of flow patterns. **a** Channel width of 150 µm. **b** Channel width of 200 µm. **c** Channel width of 300 µm. *L*, trichome length. *w*, channel width

We analysed the first 25 trichomes in each high-speed video acquired under each condition (channel width and Reynolds number) and mapped the observed flow patterns on a plane defined by the confinement ratio *L*/*w* (trichome length *L* normalized by channel width *w*) and Reynolds number (Fig. 9). Although Fig. 9 is not intended to define sharp phase boundaries, it reveals trends in how flow patterns depend on the confinement and flow rate. In particular, the pattern distribution is broadly consistent with a shear-rate-driven deformation picture. Increasing the Reynolds number and/or increasing the confinement ratio (larger *L*/*w*) tends to promote more strongly deformed or unsteady configurations. A specific discussion is provided below.

For qualitative discussion based on Fig. 9, we ordered the patterns by the apparent degree of deformation as F > B > D > A > E/G > C. Pattern F is observed primarily at high Reynolds numbers ($\gtrsim {10}^{2}$) in the narrowest channel (*w* = 150 µm), where the shear rate is high across most of the cross-section. Pattern B also appears predominantly at higher Reynolds number and/or stronger confinement (large *L*/*w*), whereas Pattern D occupies a neighbouring region at intermediate Reynolds numbers, suggesting that a balance between hydrodynamic loading (set by the shear rate) and bending flexibility is required to stabilize Pattern D. Pattern A is mainly observed at low confinement ($L/w\lesssim 1$), where trichomes can reorient with minimal bending across a broad range of Reynolds numbers, and it also appears at very low Reynolds number ($\lesssim 10$), where the hydrodynamic loading is insufficient to induce substantial deformation even for long trichomes. Notably, the self-aligned patterns (Patterns D and E), which are most useful for length-based trichome separation, are predominantly observed within an intermediate operating window of the Reynolds numbers (light-blue shaded region in Fig. 9) in narrower channels (*w* = 150 and 200 µm). Within and around this window, Pattern E tends to occur at lower confinement ratios, whereas Pattern D becomes more prevalent at higher confinement ratios, indicating that confinement plays a key role in selecting self-aligned configurations. Pattern G is observed mainly at higher Reynolds numbers in wider channels (*w* = 200 and 300 µm), which is consistent with a regime where trichomes can adopt a near-perpendicular orientation with limited bending. Because Pattern C is rarely observed, we do not further discuss its parameter dependence.

To compare the similarities and differences among channel widths more directly, we generated two supplementary plots derived from Fig. 9. Figure S2a overlays the three panels of Fig. 9 in a single plot, highlighting the overall trend described above. In contrast, Fig. S2b reorganizes the same data by Reynolds number, facilitating comparison of flow patterns among channel widths. In the highest-Reynolds-number condition examined (*Re* = 400), Pattern F appears only in the narrowest channel, whereas Pattern G appears at lower confinement ratios in the 300-µm channel than in the 200-µm channel. These channel-width-dependent differences become clearer at higher Reynolds numbers, likely because higher shear rates emphasize differences in hydrodynamic loading on the trichomes.

Overall, while these observations alone do not fully elucidate the physical origin of the self-alignment effect, they indicate that shear-rate-dependent hydrodynamic loading under confinement is an important determinant of helical-filament behaviour in microchannel flow, and they highlight a practical operating window for achieving robust outlet-based separation.

### Investigation of the device scalability

The ability to tune the trichome-length threshold for sorting may be required in future biological experiments. Our sorting experiments in this study demonstrate high purity when the length threshold is set to 300 µm. This threshold appears to be related to a geometric length scale: the arc length of a semicircle (~314 µm) whose diameter equals the distance between the upper and lower channel walls. This observation suggests that trichomes longer than 314 µm preferentially form Pattern D, whereas shorter trichomes preferentially form Pattern E, thereby enabling self-alignment-based sorting. To test whether the threshold is tunable, we examined trichome length distributions in straight channels with different widths (150 and 300 µm). As shown in Fig. S3, relative to the 200-µm-wide channel, the Pattern E population shifts towards shorter lengths in the 150-µm-wide channel, whereas it shifts towards longer lengths in the 300-µm-wide channel. These results suggest that the effective sorting threshold can be tuned by adjusting the channel width.

The role of the long straight channel also warrants discussion. In this study, we used a 42-mm straight channel for length-based sorting of *A. platensis* trichomes. Although Fig. 2 indicates that the fraction of self-aligned trichomes (Patterns D and E) increases with channel length and suggests that longer channels may improve the separation performance, the direct impact of channel length on sorting purity has not been tested. As a preliminary test, we fabricated a sorting device with a shorter straight channel (10 mm long) and performed a sorting experiment. As shown in Fig. S4, the sorting purity slightly decreased compared with that obtained using the 42-mm channel (Fig. 6). This result suggests that shortening the straight-channel length reduces the sorting performance. A plausible explanation is that a longer channel provides trichomes with more time and distance to relax toward energetically stable positions and orientations associated with Patterns D and E. We chose a straight-channel length of 42 mm so that the entire PDMS slab could be mounted on a commercially available standard microscope slide (76 mm × 26 mm). Although even longer channels could be implemented using a larger slide, this would increase the required driving pressure and could cause failure at mechanically weak points of the device, particularly the connectors between the PDMS slab and the tubing. Thus, 42 mm was selected as a practical compromise between sorting performance, device size, and mechanical robustness.

## Conclusions

In this study, we observed a consistent positional and orientational tendency of *A. platensis* trichomes under confined flow, which we refer to as a “self-alignment effect of helical filaments”: *A. platensis* trichomes align to the flow velocity profile in a straight microchannel. We applied this effect to *A. platensis* trichome sorting on the basis of trichome length by adding an expanding-channel region with five outlets. While the detailed mechanism of self-alignment remains to be elucidated, our observations suggest a reproducible flow‒structure interaction that enables practical separation. Specifically, our quantitative analysis based on high-speed camera observations indicates that the proposed method can be used to sort *A. platensis* trichomes with a high purity of >85%. Because trichome length in *A. platensis* is known to be correlated with growth stage and environmental conditions^32,33^, our length-based sorting approach may facilitate downstream biological studies of subpopulations. This platform could be applied to investigate morphological responses to stress or to improve the selection of trichomes for bioresource applications. In addition to these biological applications, our platform could be utilised for industrial applications of *Arthrospira*, such as biotemplates of metals for THz antennas^34^ and those of metal‒organic framework nanocrystals for small-scale robotics^35^.

While the detailed mechanism underlying the self-alignment of helical trichomes (Patterns D and E) remains to be fully elucidated, some of our observations can be interpreted in the context of prior microfluidic studies on particle and filament dynamics. Classical frameworks describe shear-driven rotations of rigid ellipsoids (Jeffery orbits^36^) and inertia-driven lateral migration of particles^17^. However, these rigid-particle models are likely insufficient for *A. platensis* because trichomes are deformable. Recent studies on flexible filaments in microchannels provide a more relevant basis. A simulation study^37^ reported multiple flow modes of flexible filaments, including a mode resembling Pattern D in a low-Reynolds-number regime (*Re* $\lesssim$10), and experiments with actin filaments^38^ also revealed a Pattern D-like configuration at very low Reynolds number (*Re* $\lesssim$0.01). Together with our velocity-profile analysis, these findings suggest that Pattern D arises from shear-induced hydrodynamic loading coupled with bending flexibility, which stabilizes a curved configuration. In contrast, to the best of our knowledge, an analogue of Pattern E has not been reported. Previous studies on helical (chiral) objects have mainly focused on migration/drift and chiral separation in shear/Poiseuille flows or on generalized orientation dynamics^39–42^, rather than on stable self-aligned configurations such as those observed in this article. This suggests that additional factors specific to our system, such as axial extensibility, helical geometry, and/or finite inertia effects, may contribute to forming Pattern E. Further theoretical, numerical, and experimental work will be required to establish a unified mechanism for the self-alignment phenomena reported here.

## Materials and methods

### Definition of the Reynolds number

The Reynolds number *Re* is a dimensionless parameter describing the ratio between inertial and viscous forces and is defined as $Re=\rho U{D}_{h}/{\rm{\mu }}$, where $\rho$, $U$, ${D}_{h}$, and ${\rm{\mu }}$ are the density, mean velocity, hydraulic diameter of the channel, and dynamic viscosity of the fluid, respectively. The hydraulic diameter of channel ${D}_{h}$ is given by ${D}_{h}=2{wh}/(w+h)$, where $w$ and $h$ are the channel width and height, respectively. In the experiments, the mean velocity $U$ is calculated using the flow rate $Q$ as follows: $U=Q/({wh})$.

### Simulation model

**Fig. 10:**
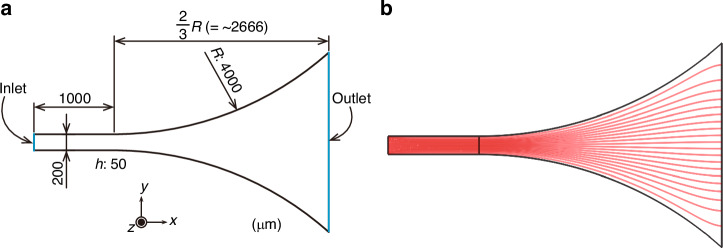
Flow simulation in the expanding-channel region. **a** Three-dimensional simulation model. Inlet: Reynolds number, 40. Outlet: pressure, 0 Pa. *h*, channel height. **b** Streamlines on the *z*-midplane

We constructed two three-dimensional simulation models in this article (Figs. 7a and 10a). In this subsection, the common settings are described. We used commercially available software COMSOL Multiphysics (version 6.4, COMSOL, USA). Both models have one inlet where a uniform inlet velocity is prescribed and one outlet where the pressure is set to 0 Pa. The inlet velocity was adjusted to achieve the target Reynolds number.

### Functionality of the expanding channel

The expanding channel serves to expand the lateral positional gap between trichomes flowing at different positions across the straight channel, increasing the separation efficiency. Therefore, uniform streamline expansion from the straight-channel region to the expanding-channel region is ideal. Generally, such ideal flow distribution is achieved in the low-Reynolds-number region. To confirm whether the Reynolds number of 40 is acceptable, we constructed a three-dimensional simulation model (see the “Simulation model” subsection in the “Materials and methods” section for the detailed settings) that mimics the channel we used in this article. Specifically, the model, as shown in Fig. 10a, consists of a 1-mm-long by 200-µm-wide straight channel and an expanding channel with a radius of 4 mm. The channel height is 50 µm. Figure 10b shows the streamlines on the *z*-midplane with the Reynolds number of 40, confirming that the streamlines are almost equally expanded.

### Microfluidic chip preparation

We prepared a microfluidic chip, a flowing-trichome observation setup, and *A. platensis* cells. The microfluidic chip was fabricated via the soft lithography process described in Ref. ^43^ as a basis. Briefly, a master mould was prepared by patterning a photoresist (SU-8 3050, MicroChem, USA) on a 4-inch silicon wafer to a thickness of 50 µm. A mixture of polydimethylsiloxane (PDMS; Sylgard 184, Dow Corning, USA) base and curing agent at a 10:1 ratio was poured onto the mould, degassed, and cured in an oven at 60 °C for 2 h. After curing, the PDMS was peeled off, and inlet and outlet holes were punched. Afterwards, the peeled PDMS and a glass substrate were exposed to UV light for 2 min (Min-Excimer SUS713, USHIO, Japan) and bonded by contacting and heating them on a hotplate at 100 °C for 30 min.

### Cell preparation

The *A. platensis* strain NIES-39 was obtained from the Microbial Culture Collection at the National Institute for Environmental Studies (NIES) and cultured in 15 mL of SOT medium in 50-mL flasks. The cells were subcultured weekly and incubated in a custom-built growth chamber at 22 °C under warm-white LED lighting with a 10-h/14-h light/dark cycle.

### Sorting operation and evaluation

The experiments we performed in this article can be classified into two groups. The first group of experiments was evaluated by high-speed imaging (Figs. 2–5, 8, and 9) while the second group of experiments involved a sorting experiment, which was evaluated using collected samples (Fig. 6).

The operation and evaluation procedures of the first group of experiments are described as follows. The cell suspension was introduced into the microfluidic channel via a syringe pump (11 Elite, Harvard Apparatus, USA). Trichome trajectories were observed via an inverted microscope (ECLIPSE Ti2, Nikon, Japan) equipped with a high-speed camera (Phantom Miro eX4, Vision Research, USA). High-speed videos were captured with an exposure time of 2 µs and a frame rate of 2900 frames per sec. The videos were analysed manually by tracking the centre positions and lengths of individual trichomes. Trichomes flowing in the microchannel were manually classified into seven flow patterns (Patterns A-G; see the “Results and discussion” section for details). Trichomes that overlapped with others were excluded from the analysis.

The purities and yields were calculated using the following equations:

$${{\mathrm{Yield}}\,{\mathrm{of}}\,{\mathrm{O}}1}=\,\frac{{{\rm{O}}1}_{{\rm{L}}}/{n}_{{\rm{O}}1}}{{\sum }_{i=1}^{3}({{\rm{O}}i}_{{\rm{L}}}/{n}_{{\rm{O}}i})},\,{{\mathrm{Yield}}\,{\mathrm{of}}\,{\mathrm{O}}}3=\,\frac{{{\rm{O}}3}_{{\rm{S}}}/{n}_{{\rm{O}}3}}{{\sum }_{i=1}^{3}({{\rm{O}}i}_{{\rm{S}}}/{n}_{{\rm{O}}i})},$$

$${{\mathrm{Purity}}\,{\mathrm{of}}\,{\mathrm{O}}1}=\frac{{{\rm{O}}1}_{{\rm{L}}}}{{{\rm{O}}1}_{{\rm{S}}}+{{\rm{O}}1}_{{\rm{L}}}},\,{{\mathrm{Purity}}\,{\mathrm{of}}\,{\mathrm{O}}3}=\,\frac{{{\rm{O}}3}_{{\rm{S}}}}{{{\rm{O}}3}_{{\rm{S}}}+{{\rm{O}}3}_{{\rm{L}}}},$$

where O*i*_S_ and O*i*_L_ denote the number of trichomes that were shorter and longer than the threshold flowing into Outlet *i* (*i* = 1, 2, or 3). n_O*i*_ denotes the number of trichomes flowing into Outlet *i*. The number of analysed trichomes flowing into each outlet varies among the outlets. These evaluations follow those described in our recent paper^44^ as a basis, but we normalised the values for the yield and purity calculations to compensate for this variation.

The operation and evaluation procedures of the second group of experiments are described as follows. The cell suspension was introduced into the microfluidic channel using a syringe pump at a flow rate of 300 µL/min (*Re* = 40). The initial 30-s sample was disposed of to avoid unwanted carryovers. Then, the collection tubes were placed, and sorted samples were collected for 7 min. Aliquots of the collected samples were deposited on glass slides and observed under an inverted microscope. The purity was calculated using the same equations as in the first group of experiments. The yield was not calculated for this sorting experiment because the normalisation used in the yield definition is not appropriate for evaluating sorting performance based on collected samples.

## Supplementary information

Supplemental material

Movie S1

Movie S2

Movie S3

Movie S4

Movie S5

## Data Availability

Supplementary Information is available: The Supplementary Information includes length distribution histograms of *A. platensis* trichomes used in Fig. 2 and movies demonstrating flowing trichomes in a straight channel, expanding-channel, and outlet region. The authors confirm that the data supporting the findings of this study are available within the main manuscript and the Supplementary Information.
