# Supplementary material for: Length-based separation of Arthrospira (Spirulina) platensis trichomes via the self-alignment effect of helical filaments in a straight microchannel
Source: Microsyst Nanoeng. 2026 May 12;12:164. doi: 10.1038/s41378-026-01302-4 (PMC13161404; doi:10.1038/s41378-026-01302-4)
Supplement: Supplementary file 1 — Supplemental material [file 41378_2026_1302_MOESM1_ESM.pdf]

Supplementary Information for:

Length-based separation of *Arthrospira (Spirulina) platensis* trichomes via the self-alignment effect of helical filaments in a straight microchannel

Kodai Hara[a] and Akihiro Isozaki[#a]

[a]*Department of Mechanical Engineering, Ritsumeikan University, Kusatsu 525-8577, Japan.*

#Corresponding author: aisozaki@fc.ritsumei.ac.jp

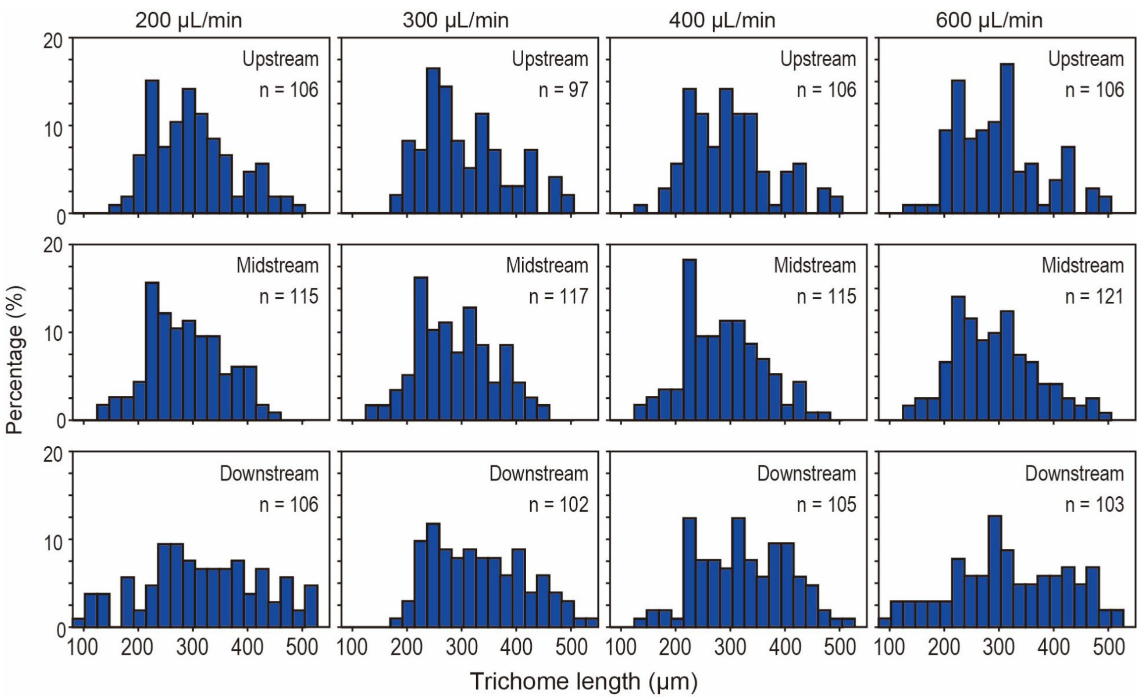

Fig. S1 Length distribution histograms of *A. platensis* trichomes used in Figs. 2 and 3.

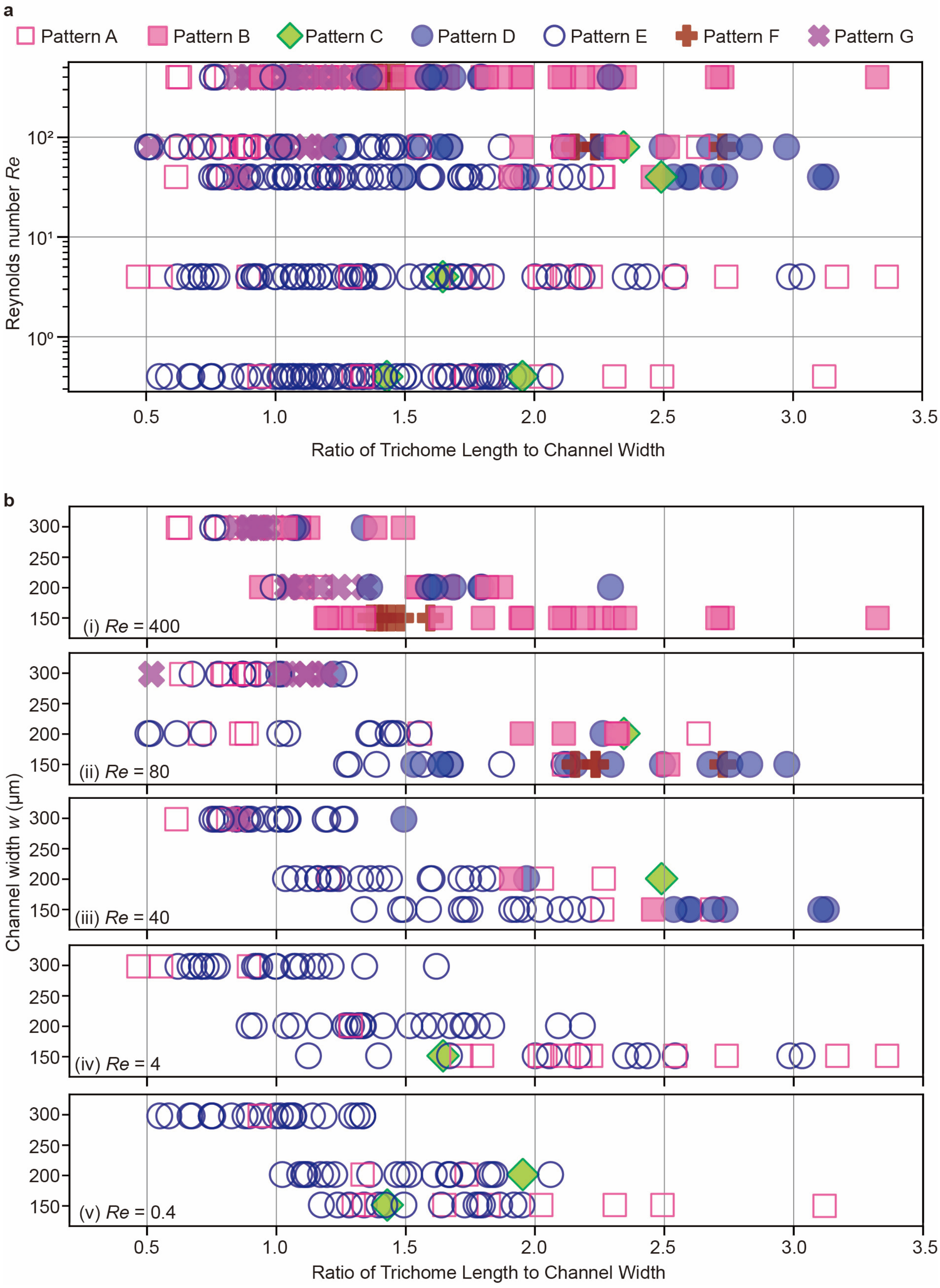

Fig. S2 Reorganized flow-pattern maps derived from Fig. 9. (a) Overlaid flow-pattern maps for the three channel widths. (b) Flow-pattern maps reorganized by Reynolds number.

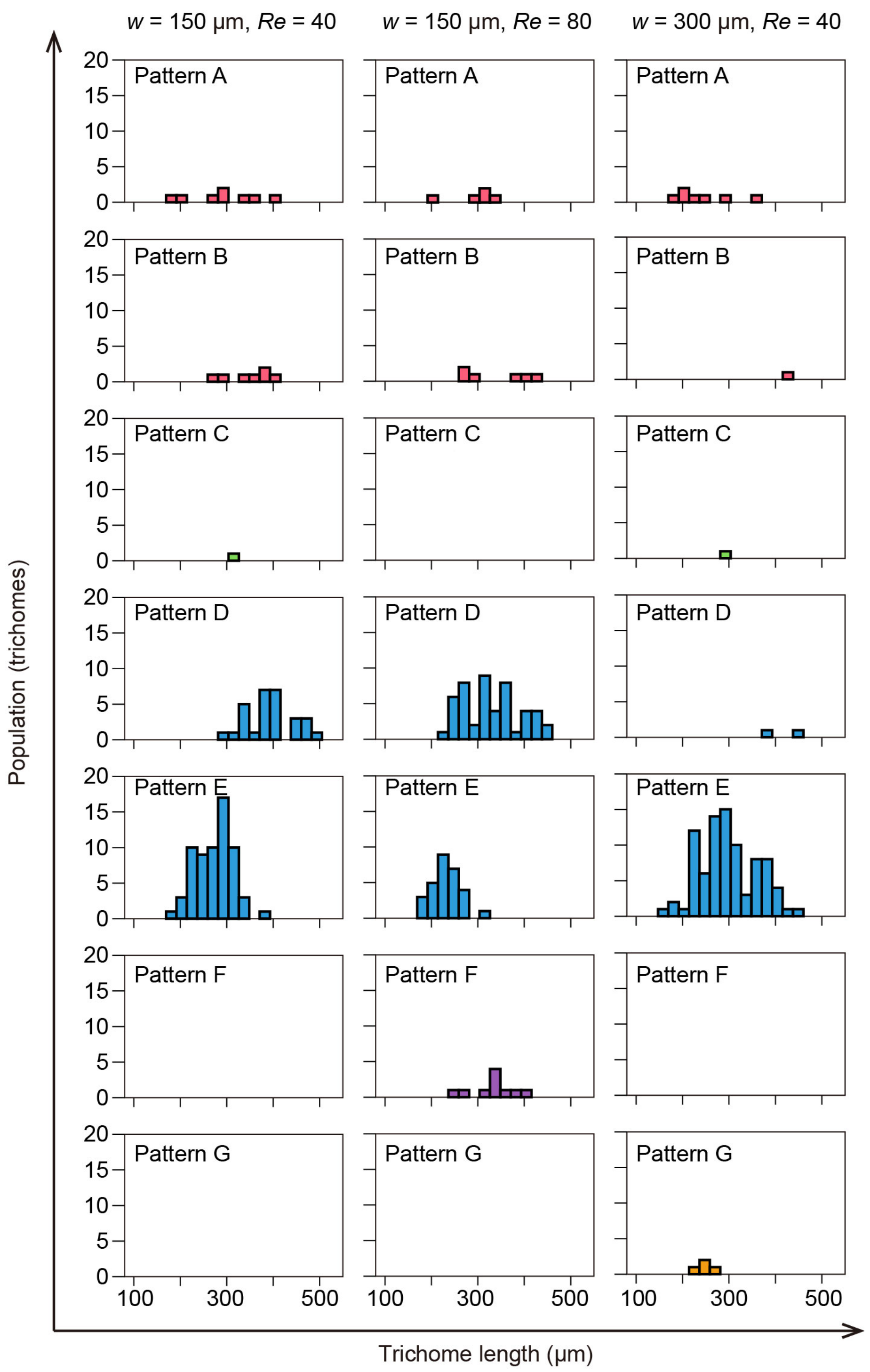

Fig. S3 Length distributions of *A. platensis* trichomes for each flow pattern in straight channels of different widths. Trichome behaviours were analysed at a downstream location (37–42 mm from the straight-channel entrance) under Reynolds numbers of 40 and 80 for a channel width of 150 µm, and under a Reynolds number of 40 for a channel width of 300 µm. Patterns A–G are defined in Figs. 1 and 8. *w*, straight-channel width. *Re*, Reynolds number.

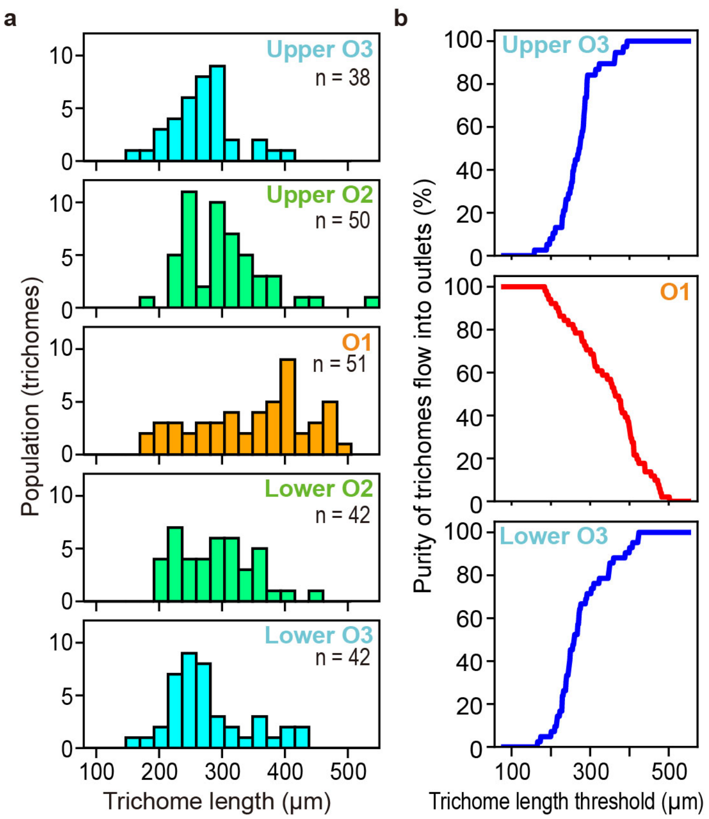

Fig. S4 Sorting demonstration using a 10-mm-long straight channel. (a) Comparison of the population of *A. platensis* trichomes collected from each outlet. (b) Purities calculated as a function of the trichome-length threshold. The Reynolds number in the straight channel was set at 40. O1, Outlet 1; O2, Outlet 2; O3, Outlet 3.
